# Complementary feeding practices and their determinants among children aged 6–23 months in rural Bangladesh: evidence from Bangladesh Integrated Household Survey (BIHS) 2018–2019 evaluated against WHO/UNICEF guideline -2021

**DOI:** 10.1186/s13690-023-01131-1

**Published:** 2023-06-21

**Authors:** Ahmed Jubayer, Abira Nowar, Saiful Islam, Md. Hafizul Islam, Md. Moniruzzaman Nayan

**Affiliations:** 1 Institute of Nutrition and Food Science, University of Dhaka, Dhaka, 1000 Bangladesh; 2 Bangladesh Institute of Social Research (BISR) Trust, Dhaka, 1207 Bangladesh

**Keywords:** Complementary feeding, CF predictors, Child feeding, Minimum dietary diversity, Bangladesh

## Abstract

**Background:**

Appropriate Complementary feeding (CF) practices play a crucial role in determining child nutrition, growth, and development. This study seeks to examine CF practices and their predictors among children aged 6 to 23 months in rural Bangladesh according to the most recently updated WHO/UNICEF guidelines for CF.

**Methods:**

A total of 665 children aged 6 to 23 months from the Bangladesh Integrated Household Survey (BIHS) 2018–2019 dataset were analyzed. The WHO/UNICEF guidelines for CF were followed to evaluate each of the nine CF practice indicators. We also examined the effect of the child, maternal, household, and community-level factors on different CF components using multiple logistic regression analyses.

**Results:**

Approximately two-thirds of the children initiated complementary feeding on time (63.5%) but had zero vegetable or fruit consumption (63.2%). More than half (52.4%) and the majority (86.5%) of children had minimum meal frequency and minimum milk feeding frequency, respectively. On the other hand, the proportion of minimum dietary diversity was quite low (18.3%), as reflected in the alarming prevalence (16.3%) of minimum acceptable diet. Egg and/or flesh food, sweet beverage, and unhealthy food consumption were 23.3%, 2.5%, and 12.2%, respectively. Child age, mothers’ education level, antenatal care visit, household food security, monthly household income, and place of residence were found to be associated with CF practices.

**Conclusion:**

When compared to results obtained using the previous guideline, the new one has resulted in a lower prevalence of Introduction of solid, semi-solid, or soft foods (ISSF), Minimum dietary diversity (MDD), Minimum meal frequency (MMF), and Minimum acceptable diet (MAD). It is crucial to convey the new knowledge for better child feeding and nutrition as the country prepares to apply the new guideline.

**Supplementary Information:**

The online version contains supplementary material available at 10.1186/s13690-023-01131-1.

**Table:** 

Text box 1. Contributions to literature
• In light of the revised WHO/UNICEF complementary feeding (CF) guideline, There is a lack of information on CF practices against all CF indicators among young children in Bangladesh.
• We assessed all the CF domains as well as their predictors as per the new guideline which may help in designing new intervention programs or pick the most relevant interventions.
• Many aspects of CF practices were very poor among rural Bangladeshi children according to new guideline. As the government prepares to implement the new indicator, it is crucial to disseminate the new information for improved child feeding and nutrition.

## Introduction

Appropriate Infant and young child feeding (IYCF) practices are widely considered as one of the most immediate solutions for child undernutrition. Optimal Complementary feeding (CF) during early infancy plays a crucial role in children’s growth, development, and survival [1]. CF is the process of initiating supplementary foods and liquids along with breast milk when breast milk alone is no longer adequate to meet an infant’s nutritional needs [2]. The introduction of energy-rich, nutrient-dense, and timely feedings fulfills the nutrition gaps of the children and can avert stunting along with under-five child mortality [3]. Moreover, the child’s growth faltering is most noticeable from 3 to 24 months, as this time is called the window of opportunity [4]. Measuring and monitoring trends in appropriate IYCF is crucial for planning programs and policies for proper child growth and development.

Bangladesh has experienced rapid economic growth in recent years. The gross national per capita income has increased to $1470, and the country is expected to become a developing country in 2026 [5]. The poverty rates have also dropped by 3.8%, and different healthcare indicators have shown significant improvements [6]. However, the poverty rate is comparatively higher in rural areas: 20.5% in rural areas and 14.7% in urban areas according to the Household Income and Expenditure Survey (HIES)- 2022 [7]. Apart from economic progress, the government of Bangladesh has emphasized reducing child undernutrition and has implemented multiple national nutrition programs and policies. Child undernutrition is still a major issue, notwithstanding the advancements in socioeconomic conditions and legislative initiatives. The prevalence of stunting and underweight among under-five children is high at 28% and 10%, respectively, whereas 23% of children are still wasted [8]. The annual reduction rate has become stagnant in the past few years, and if the current reduction rate continues (3% point per year), the prevalence of stunting and wasting among under-five children will remain at more than 25% by 2025 [9]. However, the lowest socioeconomic groups, rural areas, and slum areas have the highest rates of stunting [8, 10].

Despite Bangladesh’s above-mentioned socioeconomic improvement over the past ten years, CF practices have shown a static trend [11]. Only 28% of the young children aged 6 to 23 months received a Minimum acceptable diet (MAD) and 38% met Minimum dietary diversity (MDD) in Bangladesh according to WHO/UNICEF 2008 guideline [8, 10]. The optimum CF practice rate differs across rural and urban areas, according to MICS and BDHS. MDD and MAD rates are around 8–10% higher in urban settings [10].

Understanding the predictors of poor feeding practices would help identify the factors affecting CF, facilitate the implementation of policies and programs, and design intervention strategies. Na et al. 2018 [11] identified poverty, parental education, residence, and child age as independent predictors of poor CF practices among Bangladeshi children. Similarly, household socioeconomic condition, maternal education level, geographic location, birth order, and gender of child were shown significant associations with various CF complements [12, 13].

To aid in assessing IYCF, the World Health Organization (WHO) developed set of indicators of appropriate feeding practices for children aged 6 to 23 months in 2008 [14]. However, the previous version of the guideline has been criticized for shortcomings identified by users over the preceding decade [15]. Therefore, IYCF-2008 indicator definitions were revised in 2021, and a few new indicators were added to accommodate user demand for new information, followed by two inter-agency meetings in 2017 and 2018 [16]. This new set of indicators did not include any categorization such as core and optional as the previous guideline and recommended assessing all 17 indicators in a population. The definition of minimum dietary diversity, minimum meal frequency, and minimum acceptable diet is altered in the revised guideline. In addition, four new CF indicators are included related to egg and/or flesh food, unhealthy food, and beverage consumption.

The prior IYCF guideline has been followed in the literature on CF practices in Bangladesh [11–13, 17]. Till now, to our knowledge, no study has assessed the new CF indicators of 2021 except the one conducted by Roy et al. (2022) [17]. However, they only analyzed MDD as per the new guideline. Apart from this, child food poverty is measured based on MDD in UNICEF IYCF global database using DHS/MICS survey data [18]. In light of the revised recommendation, there is a lack of information on complementary feeding practices against all CF indicators among young children in Bangladesh. It is important to comprehensively assess all the CF domains according to the most valid and reliable indicators to design new intervention programs or pick the most relevant interventions. Addressing this knowledge gap, employing the recent WHO/UNICEF guiding principles, the present study, for the first time, evaluated CF practices for all important indicators among young children living in rural Bangladesh where malnutrition and poverty rate are more prevalent than the urban ones. It is also important to identify the predictors of CF practices separately for rural and urban areas to identify whether divergent intervention and policy strategies are required for the particular setting. Even if there is a previous guideline for CF, no other study has specifically sought to generate information on predictors of CF for rural and urban context separately. Therefore, in addition to evaluating complementary feeding practices, the current study attempted to capture the influence of the influencing factors of different CF component, particularly in the setting of rural Bangladesh employing secondary data from that region. However, since our data was limited to rural Bangladesh, we couldn’t investigate the rural–urban difference in these specific issues.

## Methods

### Study design and data source

The study was designed to explore the CF practices and associated factors among children aged 6–23 months in rural Bangladesh by analyzing the latest nationally representative data of rural Bangladesh. We used data from the third round of the Bangladesh Integrated Household Survey (BIHS) conducted between 2018 and 2019, which is representative of rural areas in each of the country’s seven administrative divisions (Barisal, Chittagong, Dhaka, Khulna, Rajshahi, Rangpur, and Sylhet) [19]. BIHS is the most comprehensive survey which provides detailed data on household food security, agricultural production and practice, dietary intake and anthropometric information of individual household members, child health, and women empowerment [20]. A total of 5605 rural households were sampled in this household-level survey using a two-stage stratified sampling procedure. Under the supervision and guidance of senior International Food Policy Research Institute (IFPRI) researchers, Data Analysis and Technical Assistance (DATA) Limited implemented the BIHS survey. An adult female household member was interviewed by a female interviewer.

### Sample selection

IYCF and antenatal care practice (ANC)-related data are accessible in a separate BIHS dataset for children under the age of two. The children between 6 to 23 months of age for whom CF practice-related data were available were the only ones included in the present analyses. In the dataset, there were 681 such cases. However, only one child was chosen randomly for the analysis in cases where households had more than one child within the age range. Finally, 665 cases that met these inclusion criteria were pulled out for the final analysis.

A different dataset was prepared for evaluating the “introduction of solid, semi-solid, or soft food” (ISSSF) indicator, which is only applicable to children between the ages of six and eight months. There were 126 children for whom ISSSF-related data were available, and no household had more than one child in the relevant age range (6–8 months). Therefore, ISSSF was evaluated for 126 children.

### Complementary feeding indicators

We followed WHO/UNICEF-2021 CF practice indicators [16]. The indicators for complementary feeding status were: Introduction of solid, semi-solid, or soft foods (ISSSF), Minimum dietary diversity (MDD), Minimum meal frequency (MMF), Minimum milk feeding frequency for non-breastfed children 6–23 months (MMFF), Minimum acceptable diet (MAD), Egg and/or flesh food consumption (EFF), Sweet beverage consumption (SWB), Unhealthy food consumption (UFC), and Zero vegetable or fruit consumption (ZVF). Definitions of the indicators and key changes from IYCF 2008 indicators are given in Supplementary table 1.

### Predictor variables

The present study considered several covariates, including child, maternal, and household or community-level characteristics. These variables were identified based on literature [11, 21, 22] and established conceptual frameworks [23]. Child characteristics included the age of the child in months, gender, and birth order. Maternal characteristics were age in year, education level, number of under-five children, receiving Antenatal care (ANC), adequacy of ANC, and delivery place. Household or community level factors included household size, household food security, primary adult decision maker, and monthly per capita household (food + non-food) expenditure, and division. Household food insecurity status was defined based on the Food insecurity experience scale (FIES). FIES raw score was calculated and a food secure household was defined as a FIES score of zero, whereas, a household FIES score of ≥ 1 was defined as food insecure [24]. Supplementary table 2 lists the detailed description of these variables.

### Statistical analysis

Both descriptive and inferential statistics were measured considering the complex survey design of the BIHS (i.e.; to adjust for sampling weight). Taylor series linearization approach was followed using IBM SPSS Statistics, version 25 software [25]. Bivariate analysis was done using Pearson’s chi-square to assess for distributional differences between groups (ISSSF [yes, no], MDD [yes, no], MMF [yes, no], MAD [yes, no], EFF [yes, no], SWB [yes, no], UFC [yes, no], and ZVF [yes, no]. Good feeding practices according to guideline were coded as “yes” for each indicator while poor feeding practices were coded as “no”. The bivariate analysis did not include MMFF since there were so few non-breastfed children in the dataset.

Variables that showed significance at *p*-values < 0.25 in bivariate analyses were considered for regression models [26]. We performed multiple logistic regression analyses to find the association between covariates and various CF components. Eight different logistic regression models were built following the stepwise method (forward entry) for eight CF indicators. We examined the underlying assumption of logistic regression models before the final model building in terms of multicollinearity and validity of the model. The regression model’s validity was examined using Pearson's goodness of fit. The model’s multicollinearity was checked using the variance inflation factor (VIF), and a VIF value greater than five was regarded as evidence of multicollinearity [27]. VIF values are presented in Supplementary table 3. *P*-values < 0.05 were used to determine whether a variable was statistically significant.

## Results

### Characteristics of the study population

The general characteristics of the study individuals are shown in Table 1. One-third (33.5%) of the children were at 6–11 months of age, and around 36% and 30.6% of them were 12–17 months and 18–23 months of age, respectively. More than half (54.5%) of them were male children. A higher portion of the mothers (62%) were at 20–30 years of age, and a similar percentage was found to complete secondary or higher education. Around 89% of women received ANC, but only 45.6% had an adequate number of ANC visits, and 48.6% had their babies in a health facility. About 60% of the households were food secure. In most families (86.2%), the adult male member held the power to make primary decisions.

**Table 1:** General characteristics of the study children aged 6–23 months, Bangladesh Integrated Household Survey, 2018–2019 (*N* = 665)

Characteristics	n (%)
**Child characteristics**	
Age (in months)	
6–11	221 (33.5)
12–17	251 (35.9)
18–23	193 (30.6)
Sex	
Male	357 (54.5)
Female	308 (45.5)
Birth order	
1–2	424 (63.3)
≥ 3	241 (36.7)
**Maternal characteristics**	
Age in year	
15–19	89 (15.4)
20–30	415 (62)
31 or more	152 (22.6)
Education	
No formal education	59 (8.6)
Primary or below	197 (29.4)
Secondary or higher	400 (61.9)
Number of under-five children	
One	375 (52.5)
Two	225 (33.4)
Three or more	65 (14.1)
Received ANC	
Yes	576 (88.8)
No	72 (11.2)
Number of antenatal visits	
Adequate	254 (45.4)
Inadequate	322 (54.6)
Delivery place	
Health facility	315 (48)
Non-health facility	341 (52)
**Household/community characteristics**	
Household size	
≤ 5	403 (49.8)
≥ 6	262 (50.2)
Household food security	
Secure	378 (59.6)
Insecure	287 (40.3)
Primary adult decision maker	
Male	557 (86.2)
Female	108 (13.8)
Monthly per capita household (food + non-food) expenditure	
< 30 USD	218 (33.4)
30–45 USD	219 (33.2)
> 45 USD	228 (33.3)
Division	
Barisal	54 (6.5)
Chittagong	138 (22.1)
Dhaka	202 (30.8)
Khulna	42 (7.5)
Rajshahi	62 (13.2)
Rangpur	61 (10.9)
Sylhet	106 (9.1)

*Abbreviation*: *ANC* Antenatal care

### CF practices among children 6–23 months of age

**Fig. 1:**
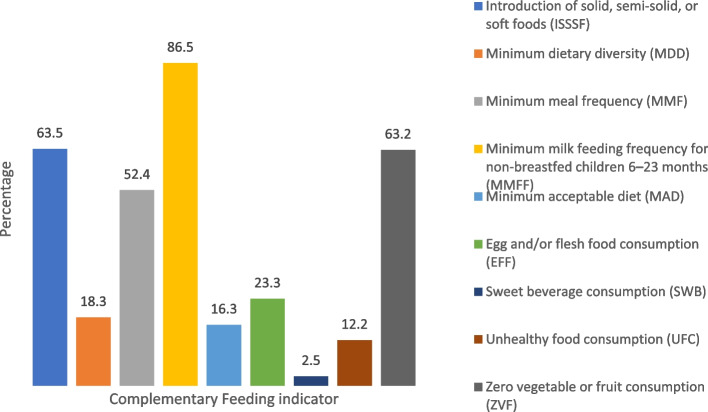
Status of complementary feeding indicators in rural Bangladesh

Percentages of children having minimum milk feeding frequency (MMFF) (86.5%), timely introduction of solid, semi-solid, or soft foods (ISSSF) (63.5%), minimum meal frequency (MMF) (52.4%) were comparatively higher compared to other indicators (Fig. 1). On the contrary, the majority of child’s mothers or primary caregivers did not give their children a minimum diverse (81.7%) and minimum acceptable diet (83.7%). Around 23% of them consumed egg and/or flesh meat, and almost 63% did not consume any fruits or vegetables the day before the study, while other indicators, SWB and UFC, were observed for 2.5% and 12.2% of children, respectively. Supplementary Table 4 further describes different CF indicators by demographic and socio-economic characteristics.

### Introduction of solid, semi-solid, or soft foods (ISSSF)

The sex of the children and their delivery place had a significant association with their timely introduction of solid, semi-solid, or soft foods (ISSF) (Table 2). The female children were two times (AOR: 2.08, 95% CI: 0.89, 4.87, *p* = 0.08) more likely to have timely ISSF compared to the male children. Similarly, the children born in health facility had two times (AOR: 2.09, 95% CI: 0.92, 4.76, *p* = 0.07) more chance to have timely ISSF compared to the children born in a non-health facility.

**Table 2:** Factors associated with ISSSF, MMF, MDD, and MAD among 6–23 months children, Bangladesh Integrated Household Survey 2018–2019

Characteristics	ISSSF aOR (95% CI)	*P* value	MDD aOR (95% CI)	*P* value	MMF aOR (95% CI)	*P* value	MAD aOR (95% CI)	*P* value
N	126		665		665		665	
Child Age (in months)								
6–11	-		0.25 (0.15, 0.4)	< 0.001	0.21 (0.12, 0.36)	< 0.001	0.2 (0.12, 0.43)	< 0.001
12–17			0.52 (0.32, 0.79)	0.003	0.41(0.24, 0.69)	0.001	0.42 (0.27, 0.65)	< 0.001
18–23 (r)			1		1		1	
Sex								
Female	2.08 (0.89, 4.87)	0.08	-		-	-	-	
Male (r)	1							
Maternal Age in year								
15–19	1.23 (0.36, 4.19)	0.73	-		0.79 (0.41, 1.99)	0.47	-	
20–30	2.04 (0.7, 4.19)	0.18			1.3 (1.82, 2.07)	0.26		
31 or more (r)	1				1			
Maternal education level								
No formal education	2.92 (0.27, 3.8)	0.37					1.03 (0.5, 2.1)	0.92
Primary or below	0.58 (0.24, 1.35)	0.2	-		-		1.7 (1.12, 2.58)	< 0.001
Secondary or higher (r)	1						1	
Received antenatal care								
No	-		0.87 (0.62, 1.29)	0.58	-		0.42 (0.22, 0.81)	0.01
Yes (r)			1				1	
Number of antenatal visits								
Inadequate	-		0.61 (0.34, 1.09)	0.09	0.84 (0.56, 1.24)	0.38	0.74 (0.51, 1.09)	0.13
Adequate (r)			1		1		1	
Delivery place								
Health facility	2.09 (0.92, 4.76)	0.07	-		-		-	
Non-Health facility (r)	1							
Household size								
≤ 5	-		-		1.12 (0.75, 1.68)	0.55	-	
≥ 6 (r)					1			
Household food security								
Insecure	-		-		0.54 (0.35, 0.81)	0.003	-	
Secure (r)					1			
Monthly per capita household expenditure								
< 30 USD	-						-	
30–45 USD			0.047 (0.3, 0.75)	0.002	0.65 (0.39, 1.09)	0.1		
> 45 USD (r)			0.69 (0.45, 1.05)	0.08	0.57 (0.36, 0.9)	0.01		
			1	1	1			
Division								
Barisal	-		0.66 (0.3, 1.41)	0.28	1.54 (0.68, 3.49)	0.29	0.76 (0.34, 1.69)	0.51
Chittagong			0.64 (0.35, 1.15)	0.14	0.65 (0.35, 1.21)	0.17	0.67 (0.36, 1.24)	0.2
Dhaka			0.95 (0.55, 1.64)	0.86	0.94 (0.52, 1.71)	0.85	0.94 (0.53, 1.67)	0.83
Khulna			2.72 (1.19, 6.2)	0.01	2.12 (0.81, 5.5)	0.13	3.67 (1.57, 8.51)	0.003
Rajshahi			0.91 (0.43, 1.83)	0.79	2.17 (0.92, 5.1)	0.07	0.79 (0.36, 1.7)	0.56
Rangpur			1.66 (0.78, 5.53)	0.18	1.73 (0.72, 4.1)	0.21	1.29 (0.59, 2.8)	0.52
Sylhet (r)			1		1		1	

*Abbreviation*: *ISSSF* Introduction of solid, semi-solid, or soft foods, *MDD* Minimum dietary diversity, *MMF* Minimum Meal Frequency, *MAD* Minimum acceptable diet, *aOR* Adjusted odds ratio

### Minimum dietary diversity (MDD)

**Fig. 2:**
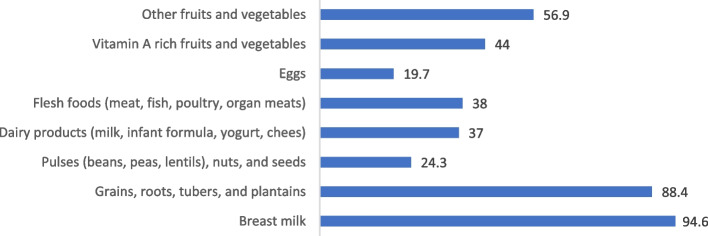
Percent distribution of children 6–23 months of age according to consumption of the eight food groups

Only about 18.3% of children aged 6–23 months were found to have at least five different food groups from eight food groups (Fig. 1). Almost all the mothers (95%) were found to feed their children with breast milk, and a higher portion of the mothers gave their children cereals (88.4%) and other fruits and vegetables (57%). However, giving eggs (19.7%), pulses (24.3%), other dairy products (37%), and flesh foods (38%) to the children were mentioned by a few mothers (Fig. 2). The age of the children, monthly per capita household expenditure, and number of ANC visits were significantly linked with the MDD of the children (Table 2). An increasing trend was found in MDD with the increment of the age of the children and monthly per capita household expenditure. Mothers who had an inadequate number of ANC visits were less likely (AOR: 0.61, 95% CI: 0.34, 1.09, p = 0.09) to give their children a minimum diversified diet compared to mothers who had an adequate number of ANC visits.

### Minimum meal frequency (MMF)

Around half of the mothers (52.4%) were found to give their children food along with breast milk a minimum number of times a day (Fig. 1). Age of the children, household food security, and monthly per capita household expenditure had significant relations with the MMF of the children (Table 2). MMF tends to be higher with the increment of the age of the children. The children from food-insecure households were less likely (AOR: 0.54, 95% CI: 0.35, 0.81, *p* = 0.003) to have MFF compared to the children from food-secure households.

### Minimum acceptable diet (MAD)

Only about 16.3% of the children were found to consume a minimum acceptable diet before the day of the survey (Fig. 1). The age of the children, the education of the mother, and antenatal care during pregnancy had a significant link with the MAD of the children (Table 2). MAD tends to be higher with the increment of the age of the children. Children of mothers having primary education were more likely (AOR: 1.7, 95% CI: 1.12, 2.58, *p* < 0.001) to have MAD compared to the children of mothers having secondary or higher education. Children of the mothers who did not receive antenatal care during pregnancy had less chance (AOR: 0.42, 95% CI: 0.22, 0.81, *p* < 0.01) to consume MAD than the children of the mothers who received antenatal care.

### Egg and/or flesh food consumption (EFF)

Less than one-fourth (23.3%) of the children consumed egg and/or flesh food (meat, fish, poultry, organ meats) during the previous day of the survey (Fig. 1). Age and sex of the children, monthly per capita household expenditure, and division were significantly linked with EFF of the children (Table 3). EFF of the children tends to be higher with the increment of the age of the children. The female children were more likely (AOR: 1.43, 95% CI: 0.99, 2.05, *p* = 0.053) to have EFF compared to the male children. Children from households with monthly per capita household expenditures < 30 USD and 30–45 USD were less likely to have EFF than those from households with monthly per capita household expenditures > 45 USD.

**Table 3:** Factors associated with EFE, SWB, UFC, and ZVF among 6–23 months children, Bangladesh Integrated Household Survey, 2018–2019

Characteristics	EFF aOR (95% CI)	*P* value	SWB aOR (95% CI)	*P* value	UFC aOR (95% CI)	*P* value	ZVF aOR (95% CI)	*P* value
N	665		665		665		665	
Child Age (in months)								
6–11	0.17 (0.1, 0.28)	< 0.001	0.09 (0.03, 2.4)	< 0.001	4.65 (2.76, 7.8)	< 0.001	7.36 (4.32, 12.2)	< 0.001
12–17	0.55 (0.35, 0.85)	0.008	0.38 (0.2, 0.7)	0.002	1.59 (1.05, 2.4)	2.6	2.13 (1.25, 3.62)	0.005
18–23 (r)	1		1		1		1	
Sex								
Female	1.43 (0.99, 2.05)	0.053	0.56 (0.31, 1.0)	0.057	-		-	
Male (r)	1		1					
Birth order								
≥ 3	0.87 (0.54, 1.4)	0.58	0.98 (0.45, 2.16)	0.97	-		-	
1–2 (r)	1		1					
Maternal Age in year								
15–19	1.71 (0.81, 3.58)	0.15	2.25 (0.69, 7.3)	0.17	-		-	
20–30	1.41 (0.81, 3,58)	0.19	1.41 (0.57, 3.5)	0.46				
31 or more (r)	1		1					
Maternal education level					-		-	
No formal education			0.42 (0.08, 2.1)	0.08				
Primary or below			1.78 (0.91, 3.4)	0.027				
Secondary or higher (r)			1					
Received antenatal care								
No	0.71 (0.4, 1.2)	0.26	0.77 (0.25, 2.38)	0.65	1.56 (0.79, 3)		-	
Yes (r)	1		1		1			
Number of antenatal visits								
Inadequate	0.9 (0.62, 1.31)	0.58	1.79 (0.98, 3.2)	0.054	-		1.26 (0.84, 1.87)	0.24
Adequate (r)	1		1				1	
Delivery place								
Health facility	-		0.57 (0.3, 1.07)	.08	-		-	
Non-health facility (r)			1					
Household food security								
Insecure	-		-		-		1.67 (1.12, 2.49)	0.012
Secure (r)							1	
Monthly per capita household expenditure								
< 30 USD								
30–45 USD	0.61 (0.37, 0.98)	0.044	0.39 (0.16, 0.9)	0.029	1.28 (0.8, 2.03)	0.3	-	
> 45 USD (r)	0.6 (0.38, 0.91)	0.023	0.46 (0.24,0.89)	0.021	1.35 (0.87, 2.1)	0.18		
	1		1		1			
Division								
Barisal	2.2 (1.03, 4.69)	0.04	1.015 (0.3, 3.1)	0.98			1.38 (0.63, 3.04)	0.41
Chittagong	1.16 (0.64, 2.09)	0.63	0.81 (0.35, 1.85)	0.62	-	-	1.92 (1.02, 3.58)	0.04
Dhaka	1.29 (0.73, 2.26)	0.37	0.33 (0.13, 0.81)	0.01			1.53 (0.84, 2.78)	0.16
Khulna	8.98 (3.3, 24.45)	< 0.001	0.79 (0.22, 2.76)	0.7			0.4 (0.14, 1.14)	0.08
Rajshahi	1.59 (0.75, 3.39)	0.22	0.23 (0.05, 0.93)	0.04			0.73 (0.32, 1.68)	0.47
Rangpur	2.33 (1.06, 5.12)	0.03	0.29 (0.05, 1.5)	0.14			0.4 (0.17, 1.17)	0.1
Sylhet (r)	1		1				1	

*Abbreviation*: *EFF* Egg and/or flesh food consumption, *SWB* Sweet beverage consumption, *UFC* Unhealthy food consumption, *ZVF* Zero vegetable or fruit consumption, *aOR* Adjusted odds ratio

### Sweet beverage consumption (SWB)

An insignificant portion (2.5%) of the children (6–23 months of age) consumed a sweet beverage during the previous day (Fig. 1). Age, sex of the children, educational level of the mother, number of ANC visits, delivery place, monthly expenditure of the household, and division had significant associations with SWB of the children (Table 3). SWB tends be higher with the increment of the age of the children and their monthly expenditure of the household. The female children were less likely (AOR: 0.56, 95% CI: 0.31, 1.0, *p* = 0.057) to have SWB compared to the male children. Children of mothers having an inadequate number of ANC had less chance (AOR: 1.79, 95% CI: 0.98, 3.2, *p* = 0.054) to have SWB than the children of mothers having an adequate number of ANC. Children born in a place with a health facility were less likely (AOR: 0.57, 95% CI: 0.3, 1.07, *p* = 0.08) to have SWB compared to children born in a place with a non-health facility.

### Unhealthy food consumption (UFC)

Only about 12.2% of the children took unhealthy foods (sugar confections, frozen treats, baked or fried confections, etc.) during the previous day (Fig. 1). Among background characteristics, only the age of the children had significant relation with their unhealthy food consumption (UCF) (Table 3). Younger children (6–11 months) had more chances (AOR: 4.65, 95% CI: 2.76, 7.8, *p* < 0.001) to consume unhealthy foods than comparatively older children (18–23 months).

### Zero vegetable or fruit consumption (ZVF)

Around 63.2% of the children (6 to 23 months of age) did not consume any vegetable or fruit during the previous day of the survey (Fig. 1). The children's age and household food security were significantly linked with zero vegetable or fruit consumption (ZVF) (Table 3). Children from food-insecure households were more likely (AOR: 1.67, 95% CI: 1.12, 2.49, *p* = 0.012) to consume no vegetables or fruit. ZVF tends to be lower with the increment of the age of the children.

## Discussion

Although Bangladesh has made remarkable progress in recent years to improve child and maternal health, child and maternal undernutrition remain a major public health concern. Despite the country’s impressive socioeconomic development, complementary feeding practices among younger children were found to be stagnant or deteriorating in a recent study [11]. Utilizing a nationally representative dataset, the current study examined the CF practices according to WHO/UNICEF new guidelines among children under two years of age.

Timely introduction of solid, semi-solid, or soft foods at six to eight months (ISSSF) is associated with a lower risk of stunting (i.e., low height in relation to a child’s age) and underweight (low weight for a given age of a child) [1]. We found that 63.5% of rural Bangladeshi infants consumed solid, semi-solid, or soft foods during the previous day, which was lower than the latest Bangladesh Demographic and Health Survey (BDHS) [10]. Studies conducted in Nepal (53.3%) [22] and Afghanistan (56%) [28] showed lower ISSSF rates than those found in the current study in rural Bangladesh. We found no significant association with any demographic or socioeconomic factors, most likely due to the small sample size (n = 126 for ISSSF indicator).

Between the ages of 6 and 23 months, feeding children a variety of foods will help them get all the nutrients they need. Minimum dietary diversity serves as a proxy for adequate micronutrient density of foods [2], and lack of dietary diversity can increase the risk of micronutrient deficiencies, which could be detrimental to physical and cognitive development [29]. Only 18.3% of infants in the current study received a minimum diversified diet, which is less than half of the latest national survey [10]. This is because the revised recommendation contains eight food groups, while the BDHS/MICS estimates were based on only seven. Consistent with a previous study in Bangladesh [11], the current study also revealed that children ages 6–11 months were 75% and children ages 12–17 months were 48% less likely to meet MDD compared to the older age group. Similar findings were observed in studies conducted in Malawi [21], Ethiopia [30], and India [31]. Similar to a recent study conducted in Malawi [21], we found that children from poorer households had a lower likelihood of having an adequate diversified diet than children from wealthier households. Richer households are more likely to be food secure and able to afford a wider variety of food items.

The prevalence of MMF was observed in 52.4% of the children. This estimate is appreciably lower than what was previously reported; around 81% of children had been fed the minimum number of times, according to the latest BDHS survey [10]. The most reasonable explanation behind this discrepancy is the alteration in the definition of this indicator in our study. Because the definition of MMF has been updated in the revised guideline (Supplementary Table 1), our data cannot be directly compared to those of research that followed the prior standard. When defining MMF, previous guidelines solely included breastfeeding; the 2021 guideline expands this to include non-milk feeding. Compared to older children, younger children had lower odds of receiving MMF. This is inconsistent with a study conducted in Malawi [21] but corroborates findings observed in studies conducted in Ethiopia [32], Ghana [33], and Sri Lanka [34]. In our analysis, children from food-insecure households and with low household monthly income had a lower likelihood of receiving MMF—a finding consistent with existing literature from Bangladesh [11].

Only 16.3% of children had been served a minimum acceptable diet, which is less than half of the BDHS estimation (35%) [10]. Our data cannot be directly compared to BDHS because the indicator definition, like MMF, was somewhat modified. Similar to Na et al. 2018 [11] findings, younger children had lower odds of meeting MAD. Surprisingly, our study showed that, compared to children of mothers with secondary or higher education, children of those with primary education or below had higher odds of meeting MAD. This is inconsistent with Na et al. 2018 [11] study conducted in Bangladesh. Although child mothers possessed academic knowledge, it is possible that they lacked understanding regarding optimal complementary feeding practices. The present analysis also revealed the positive role of ANC in feeding children adequately, which is consistent with another study [35].

According to the WHO’s guiding principles, “meat, poultry, fish, or eggs should be consumed daily, or as often as possible,” for breastfed and non-breastfed children [2]. Egg consumption is linked to higher calorie, protein, essential fatty acid, vitamin B_12_, vitamin D, phosphorus, and selenium intakes, as well as higher recumbent length [36]. Evidence also suggests that the introduction of meat as an early complementary food improves protein and zinc intake [37]. In the present analysis, 23.3% of children received egg and/or flesh food (EFF) during the previous day. We couldn’t compare the MMF to another study because it was recently added to the updated guideline. However, the proportion of children who ate eggs was substantially lower than what the BDHS estimated (19.7% vs. 29.2%) [10]. On the other hand, the percentage of children that consume flesh food in the current survey is nearly identical to that which was reported in BDHS. Similar to other indicators, children from poorer households had lower odds of meeting EFF, which could be explained by the fact that egg and flesh foods are more affordable in affluent families.

Sweet drinks only provide energy and no additional nutrition. Children of all ages are more likely to become obese when they consume more sugar-sweetened beverages. The introduction of sugar-sweetened beverages at earlier ages (before 12 months of age) is associated with obesity at six years of age [38]. Free sugars, including 100% fruit juice and sugar-sweetened beverages, also increase the risk of dental caries [39]. SWB is a new CF indicator, and it was estimated at 2.5% for rural Bangladeshi infants. Children of mothers with less than a primary level of education were more likely to consume sugar-sweetened beverages than those with a secondary or higher level of education. Education may positively impact food choices because it makes mothers more concerned about their children’s health and more likely to steer clear of unhealthy foods. Apart from that, children from low-income families were less likely to consume sugar-sweetened beverages. The possible reason may be the relatively higher cost of sweetened beverages like fruit juices and candy.

Another new indicator in the updated guideline is Unhealthy food consumption (UFC). With the upward socioeconomic condition, consumption pattern is also changing, and intakes of added sugars, unhealthy fats, salt, and refined carbohydrates are increasing in low- and middle-income countries. Consuming these foods could limit the intake of vital vitamins and minerals and replace them with less nutritious foods. Moreover, consuming unhealthy snacks and beverages has been linked to a higher risk of nutrient deficiency and shorter length-for-age [40]. Besides, childhood exposure to sweet foods and drinks regularly may develop an innate preference for sweetness, leading to increased consumption of sweet-tasting foods and drinks as a later-learned preference [41]. This study found that 12.2% of cases involved unhealthy eating habits, indicating the need to educate rural residents about child-feeding practices.

Zero vegetable or fruit consumption (ZVF) is also a new indicator in the revised guideline. Low vegetable and fruit consumption is one of the dominant drivers of non-communicable diseases. Grimm et al. 2014 [42] also found that a young child’s low fruit and vegetable intake is related to lower intake in later life. Approximately 63% of rural Bangladeshi children had not consumed any fruits or vegetables (ZVF) in the previous 24 h, with the prevalence of ZVF being much higher among younger children. High ZVF prevalence suggests that special focus should be placed on raising awareness regarding the significance of consuming fruits and vegetables in early life.

### The implication of the study findings

Using a nationally representative dataset, the current study examined the various aspects of CF practices among children aged 6 to 23 months in rural Bangladesh. Our analyses suggest that policies, programs, and initiatives should prioritize younger children (≤ 17 months) because they are more likely to be fed inappropriately in terms of the majority of the CF indicators. Certain CF indicators, such as MAD, highlight the necessity of nutrition and/or adequate infant feeding practice knowledge among mothers, notwithstanding better academic knowledge. When planning nutrition programs, policymakers should adopt an integrated approach incorporating other fields like nutrition behavior and social change education. Moreover, strategies for improving access to nutritious foods need to be coupled with nutrition projects, as this study revealed low household monthly income and food insecurity as risk factors for poor CF practices in rural Bangladesh. We also found poor dietary diversity, which points to the need to encourage locally sourced foods that are both affordable and high in nutrients. To sum up, contextually tailored (i.e., for poorly educated caregivers and low-income households) nutrition education programs should communicate the importance of appropriate CF to mothers/caregivers along with strategies for availing adequate complementary foods using households’ limited resources to improve child feeding practices.

## Strength and limitations

BIHS is a household-based survey collecting information on various agriculture, nutrition, and development issues. Questions related to complementary feeding indicators were applicable to those households where there was at least one child less than 2 years of age. The number of eligible cases was only 665, therefore, we can’t generalize the findings for the entire rural Bangladesh. The sample size for measuring some of the CF indicators was low in the original datasets, which may limit the study’s power since the power of the study increases with the sample size [43]. Additionally, due to the extremely low number of non-breastfed children, we were unable to disaggregate our analysis between breastfed and non-breastfed children. The predictive analysis could not be carried out for MMFF for the same reason. In addition, a causal inference couldn’t be drawn due to the cross-sectional nature of the study. We were unable to include other variables that might have explained our outcome variables because we used secondary data. We identified a wide range of CF predictors that have practical implications for policymakers when developing suitable nutrition projects.

## Conclusion

Four of the nine CF indicators (ISSF, ISSF, MDD, MMF, and MAD) were found in various national surveys and published publications, as recommended by the prior guideline. Poorer feeding practice for these four CF components can be attributed to the adoption of the revised guideline. We also observed unhealthy feeding practices among approximately 12% of children, with 2.5% consuming sugary beverages. A comparatively higher proportion of children get appropriate feeding in terms of ISSF (63.2%) and MMF (52%). Eggs were only given to 19.7% of children, and most of them were fed cereal-based staples such as grains, roots, and tubers. Overall, child age, education level of mothers, ANC, household food security, monthly household income, and residential area were significantly associated with various components of complementary feeding practice. Quality counseling to child mothers or primary caregivers, as well as persuasive behavioral change advocacy to other family and community decision-makers, with an emphasis on younger children, may be an effective strategy to enhance complementary feeding practice.

## Supplementary Information

**Additional file 1: Supplementary Table 1. **Definition of complementary feeding practice indicators and changes. **Supplementary Table 2.** List of predictors. **Supplementary Table 3. **Variance Inflation factorsamong the explanatory variables. **Supplementary Table 4. **Complementary feeding practice by background characteristics among children6-23 months, rural Bangladesh, 2018-2019.

## Data Availability

BIHS datasets are available online at https://dataverse.harvard.edu/dataset.xhtml?persistentId=doi:10.7910/DVN/NXKLZJ.

## Abbreviations

AOR: Adjusted Odds Ratio
ANC: Antenatal care
BIHS: Bangladesh Integrated Household Survey
BDHS: Bangladesh Demographic and Health Survey
CF: Complementary feeding
EFF: Egg and/or Flesh Food Consumption
IFPRI: International Food Policy Research Institute
ISSSF: Introduction of solid, semi-solid, or soft foods
IYCF: Infant and Young Child Feeding
MAD: Minimum Acceptable Diet
MDD: Minimum Dietary Diversity
MMF: Minimum Meal Frequency
MICS: Multiple Indicator Cluster Survey
MMFF: Minimum Milk Feeding Frequency for Non-breastfed Children 6–23 Months
SWB: Sweet Beverage Consumption
UFC: Unhealthy Food Consumption
WHO: World Health Organization
VIF: Variance inflation factor
ZVF: Zero Vegetable or Fruit Consumption
