# Supplementary material for: Complementary feeding practices and their determinants among children aged 6–23 months in rural Bangladesh: evidence from Bangladesh Integrated Household Survey (BIHS) 2018–2019 evaluated against WHO/UNICEF guideline -2021
Source: Arch Public Health. 2023 Jun 21;81:114. doi: 10.1186/s13690-023-01131-1 (PMC10286447; doi:10.1186/s13690-023-01131-1)
Supplement: Supplementary file 1 — Additional file 1: Supplementary Table 1. Definition of complementary feeding practice indicators and changes. Supplementary Table 2. List of predictors. Supplementary Table 3. Variance Inflation factorsamong the explanatory variables. Supplementary Table 4. Complementary feeding practice by background characteristics among children6-23 months, rural Bangladesh, 2018-2019. [file 13690_2023_1131_MOESM1_ESM.docx]

**Supplementary table 1: Definition of complementary feeding practice indicators and changes**

| **Indicator** | **Age group** | **Definition/measurement technique** | **Key change from 2008 definition** |
| --- | --- | --- | --- |
| Introduction of solid, semi-solid, or soft foods (ISSSF) | 6 to 8 months | “Percentage of infants six to eight months of age who consumed solid, semisolid, or soft foods during the previous day”. | No changes |
| Minimum dietary diversity (MDD) | 6-23 months | Eight food groups were considered to calculate the dietary diversity score of infants 6-23 months of age: breast milk; grains, roots, tubers, and plantains; pulses (beans, peas, lentils), nuts and seeds; dairy products (milk, infant formula, yogurt, cheese); flesh foods (meat, fish, poultry, organ meats); eggs; vitamin-A rich fruits, and vegetables; other fruits and vegetables. Mothers or primary caregivers of children were asked if their children had eaten these foods in the preceding 24 hours. “Yes” responses were coded as 1, and “No” responses were coded as 0. For analytical purposes, “refused” or “don’t know” were considered missing values. After that, the dietary diversity score was calculated by summing up the reported number of food groups consumed. According to the new WHO/UNICEF definition, MDD was defined as the “percentage of children 6–23 months of age who consumed foods and beverages from at least five out of eight defined food groups during the previous day”. | Breast milk added as an eighth food groups and cut-off for MDD increased to five food groups. |
| Minimum meal frequency (MMF) | 6 to 23 months | MMF was defined as the “percentage of children 6–23 months of age who consumed solid, semi-solid, or soft foods (but also including milk feeds for non-breastfed children) at least the minimum number of times during the previous day.” The number of times was defined as twice for breastfed infants aged six to eight months, three times for breastfed children aged 9-23 months, and four feedings of solid, semi-solid, or soft foods or milk feeds for non-breastfed children aged 6–23 months whereby at least one of the four feeds must be a solid, semi-solid, or soft feed. | At least one non-milk feeding is required to meet minimum for non-breastfed children while the previous definition allowed children to achieve the minimum with milk feeds only |
| Minimum milk feeding frequency for non-breastfed children 6–23 months (MMFF) | 6 to 23 months | MMFF was defined as the “percentage of non-breastfed children 6–23 months of age who consumed at least two milk feeds during the previous day.” Any formula (such as infant formula) or animal milk (other than human breast milk), as well as semi-solid and fluid/drinkable yogurt, were considered as milk feeds. | This was an “optional” indicators |
| Minimum acceptable diet (MAD) | 6 to 23 months | MAD was defined as the “percentage of children 6–23 months of age who consumed a minimum acceptable diet during the previous day”. The definition of a minimum acceptable diet was different for breastfed and non-breastfed children. Breastfed children who had MDD and MMF for their age were defined as MAD. On the other hand, for non-breastfed children, MAD was defined as receiving MDD and MMF for their age, and at least two milk feeds during the previous day. | Altered to reflect changes in MDD and MMF above |
| Egg and/or flesh food consumption (EFF) | 6 to 23 months | EFF was defined as the “percentage of children 6–23 months of age who consumed egg and/or flesh food (meat, fish, poultry, organ meats) during the previous day.” | New indicator |
| Sweet beverage consumption (SWB) | 6 to 23 months | SWB was defined as the “percentage of children 6–23 months of age who consumed a sweet beverage during the previous day.” | New indicator |
| Unhealthy food consumption (UFC) | 6 to 23 months | UFC was defined as the “percentage of children 6–23 months of age who consumed selected sentinel unhealthy foods during the previous day”. Sentinel unhealthy foods included candies, chocolate, and other sugar confections; Frozen treats like ice cream, sorbet, cakes, pastries, sweet biscuits, and other baked or fried confections; chips, French fries, instant noodles, and similar items. | New indicator |
| Zero vegetable or fruit consumption (ZVF) | 6 to 23 months | ZVF was defined as the “percentage of children 6–23 months of age who did not consume any vegetables or fruits during the previous day.” | New indicator |

**Supplementary table 2: List of predictors**

| **Variable** | **Categories** |
| --- | --- |
| **Child characteristics** | |
| Age of the child ( in months) | 6 -11, 12-17, 18-23 |
| Gender | Male, female |
| Birth order | 1-2, ≥3 |
| **Maternal characteristics** | |
| Age in year | 15-19, 20-30, 31 or above |
| Education level | No formal education, primary or below, secondary or higher |
| Number of under-5 children | one, two, three or more |
| Receive Antenatal care (ANC) | Yes, no |
| The status of ANC | Adequate, inadequate |
| Delivery place | health facility, non-health facility |
| **Household or community level factors** | |
| Household size | ≤5, ≥6 |
| Household food security | Food secure, food insecure |
| Primary adult decision maker | Male, female |
| Monthly per capita household (food+non-food) expenditure | <30 USD, 30-45 USD, >45 USD |
| Division | Barisal, Chittagong, Dhaka, Khulna, Rajshahi, Rangpur, and Sylhet |

**Supplementary Table 3: Variance Inflation factors (VIF) among the explanatory variables**

| **Variable** | **VIF** |
| --- | --- |
| Age of child  12 to 17  18 to 23 | 1.39  1.42 |
| Gender of child  Female | 1.03 |
| Birth order  ≥3 | 1.69 |
| Maternal Age in year  15-19  20-30 | 2.28  2.89 |
| Maternal education level  Primary or below  Secondary or higher | 3.75  4.2 |
| Number of U-5 children  More than one | 1.19 |
| Received antenatal care  No | 1.0 |
| Number of antenatal visits  Inadequate | 1.16 |
| Delivery place  Health facility | 1.23 |
| Household size  ≤5 | 1.12 |
| Household food security  Insecure | 1.15 |
| Monthly per capita household expenditure  <30 USD  30-45 USD | 1.67  1.35 |
| Division  Barisal  Chittagong  Dhaka  Khulna  Rajshahi  Rangpur | 1.5  2.07  2.23  1.43  1.57  1.57 |

**Supplementary table 4: Complementary feeding practice by background characteristics among children 6-23 months, rural Bangladesh, 2018-2019**

| Characteristics | ISSF  Yes, n (%) | MDD  Yes, n (%) | MMF  Yes, n (%) | MMFF  Yes, n (%) | MAD  Yes, n (%) | EFF  Yes, n (%) | SWB  Yes, n (%) | UFC  Yes, n (%) | ZF  Yes, n (%) |
| --- | --- | --- | --- | --- | --- | --- | --- | --- | --- |
| Total | 81 (63.5) | 19 (18.3) | 67 (52.4) | 32 (86.5) | 17 (16.3) | 27 (23.3) | 4 (2.5) | 13 (12.2) | 79 (63.2) |
| **Child characteristics** |  |  |  |  |  |  |  |  |  |
| Age (in months)  6-11  12-17  18-23 | NA | 50 (24.5)  99 (39.9)  107 (57.8) | 121 (55.7)  172 (68)  159 (82.9) | - | 39 (19.9)  80 (31.6)  92 (48.4) | 60 (28.1)  133 (53.9)  129 (68.7) | 6 (2)  24 (9.6)  43 (20.5) | 24 (12.1)  65 (26)  71 (38.9) | 116 (53.4)  69 (25.2)  30 (14.6) |
| Sex  Male  Female | 46 (55.1)  35 (78.7) | 134 (38)  122 (43.2) | 243 (68.6)  208 (68.3) | - | 115 (32.2)  96 (33.6) | 157 (45.3)  165 (55.7) | 45 (12.9)  28 (7.7) | 86 (25.4)  74 (25.4) | 112 (31.2)  102 (31.2) |
| Birth order  1-2  ≥3 | 57 (66.8)  24 (56.2) | 166 (41.5)  38 (38.7) | 289 (68.4)  162 (68.9) | - | 137 (33.7)  74 (31.6) | 221 (54)  101 (43.6) | 52 (11.6)  21 (8.6) | 105 (25.8)  55 (24.9) | 142 (33)  72 (28.3) |
| **Maternal characteristics** |  |  |  |  |  |  |  |  |  |
| Age in year  15-19  20-30  31 or more | 16 (59)  52 (68)  13 (55.6) | 33 (44.4)  163 (40.3)  58 (38.7) | 54 (61.4)  289 (69.5)  100 (68.6) | - | 27 (34.5)  135 (33.2)  48 (31.9) | 46 (65.7)  207 (51.1)  63 (41.1) | 12 (12.4)  48 (10.7)  11 (7.5) | 20 (23.8)  107 (26.4)  32 (23.8) | 34 (36.5)  127 (30.3)  51 (30.6) |
| Education  No formal education  Primary or below  Secondary or higher | 6 (88.9)  21 (54.8)  54 (65.8) | 22 (41.1)  84 (42.1)  148 (39.8) | 40 (72.8)  132 (66.5)  271 (68.1) | - | 20 (37.9)  73 (35.8)  117 (31.1) | 23 (43.5)  99 (49.4)  194 (50.7) | 2 (3.2)  23 (11.2)  46 (10.8) | 11 (22.2)  55 (27.7)  93 (24.8) | 14 (20.5)  66 (31.3)  132 (32.8) |
| Number of U-5 children  More than one  One | 40 (66.6)  41 (60.5) | 112 (40.8)  144 (40) | 198 (69)  253 (67.9) | - | 89 (31.8)  122 (33.7) | 132 (47.8)  190 (52) | 29 (9.6)  44 (11.3) | 72 (26.6)  88 (24.4) | 86 (29.4)  128 (32.8) |
| Received antenatal care  Yes  No | 69 (64.6)  12 (73.8) | 234 (41.4)  20 (32.2) | 398 (69.8)  47 (67.8) | - | 196 (34.8)  16 (26.7) | 290 (50.6)  26 (40.8) | 68 (10.9)  4 (5.7) | 147 (26.1)  12 (20.6) | 184 (30.9)  29 (35.6) |
| Number of antenatal visits  Inadequate  Adequate | 36 (57.3)  31 (71.8) | 123 (39.6)  111 (43.6) | 215 (67.9)  183 (72.2) | - | 100 (32.6)  95 (37.4) | 151 (48.6)  139 (53.1) | 44 (13.4)  24 (7.9) | 77 (25.5)  70 (26.8) | 111 (33.3)  73 (28) |
| Delivery place  Health facility  Non- Health facility | 41 (74.9)  40 (59) | 131 (40.6)  125 (40.1) | 241 (72.2)  211 (67.1) | - | 109 (33.9)  102 32.9) | 163 (49.6)  159 (50.4) | 33 (9.3)  40 (11.7) | 88 (27.9)  72 (22.8) | 113 (30.8)  102 (31.7) |
| **Household/community characteristics** | |  |  |  |  |  |  |  |  |
| Household size  ≤5  ≥6 | 49 (66.4)  32 (61.3) | 151 (37.9)  105 (42.9) | 283 (71.5)  168 (65.3) | - | 126 (31.1)  85 (34.5) | 201 (50.6)  121 (49.4) | 42 (9.4)  31 (11.6) | 92 (23.2)  68 (27.7) | 137 (32.5)  77 (29.9) |
| Household food security  Secure  Insecure | 38 (61.4)  43 (66.4) | 147 (40.7)  109 (40) | 267 (71.4)  190 (66.4) | - | 114 (31.7)  97 (34.5) | 188 (51.2)  134 (48.3) | 44 (10.9)  29 (9.8) | 93 (26.4)  67 (24) | 132 (33.4)  82 (27.8) |
| Monthly per capita household expenditure  <30 USD  30-45 USD  >45 USD | 25 (65)  25 (61.2)  31 (64.8) | 66 (32.2)  89 (43.6)  101 (45) | 146 (69.3)  140 (64.2)  165 (71.7) | - | 59 (28.2) 70 (33.2)  82 (37.1) | 90 (44)  106 (50.3)  126 (55.6) | 14 (6.6)  20 (8.8)  39 (15.9) | 45 (22.9)  50 (24.6)  65 (28.7) | 61 (25.2)  76 (32.7)  77 (35.6) |
| Division  Barisal  Chittagong  Dhaka  Khulna  Rajshahi  Rangpur  Sylhet | 8 (71.6)  13 (72.4)  18 (55.6)  5 (73)  10 (58.9)  12 (62.9)  15 (72.7) | 16 (29.9)  49 (37.9)  78 (38.7)  25 (61. 6)  22 (35.5)  28 (51.3)  38 (36.7) | 40 (69.7)  86 (62.6)  132 (65.8)  32 (80.6)  49 (78.5)  45 (71.4)  67 (62.4) | - | 14 (25.9)  37 (28.3)  63 (30.6)  23 (58.3)  18 (29.9)  24 (41.2)  32 (29.8) | 29 (51.7)  69 (51.6)  91 (45.6)  32 (77.3)  31 (46.5)  32 (55.3)  38 (36) | 6 (11.5)  25 (18.1)  15 (7.2)  5 (11.4)  5 (7)  3 (5.5)  14 (12.4) | 11 (19.1)  40 (31.7)  39 (19.8)  14 (32.2)  15 (24.7)  17 (30.2)  24 (23.7) | 18 (34)  52 (37.4)  74 (35.4)  6 (13.7)  17 (27.3)  10 (17.1)  37 (35) |

Abbreviation: ISSSF: Introduction of solid, semi-solid or soft foods; MDD: Minimum dietary diversity; MMF: Minimum Meal Frequency; MMFF: Minimum milk feeding frequency for non-breastfed children 6–23 months; MAD: Minimum acceptable diet; EFF: Egg and/or flesh food consumption, SWB: Sweet beverage consumption; UFC: Unhealthy food consumption; ZF: Zero vegetable or fruit consumption.
